# A Literature Review and Framework Proposal for Halitosis Assessment in Cigarette Smokers and Alternative Nicotine-Delivery Products Users

**DOI:** 10.3389/froh.2021.777442

**Published:** 2021-12-10

**Authors:** Filippo Zanetti, Tanja Zivkovic Semren, James N. D. Battey, Philippe A. Guy, Nikolai V. Ivanov, Angela van der Plas, Julia Hoeng

**Affiliations:** PMI R&D, Philip Morris Products S.A., Neuchâtel, Switzerland

**Keywords:** halitosis, cigarette smoke, reduced risk products, electronic vapor product, heated tobacco product

## Abstract

Halitosis is a health condition which counts cigarette smoking (CS) among its major risk factors. Cigarette smoke can cause an imbalance in the oral bacterial community, leading to several oral diseases and conditions, including intraoral halitosis. Although the best approach to decrease smoking-related health risks is quitting smoking, this is not feasible for many smokers. Switching to potentially reduced-risk products, like electronic vapor products (EVP) or heated tobacco products (HTP), may help improve the conditions associated with CS. To date, there have been few systematic studies on the effects of CS on halitosis and none have assessed the effects of EVP and HTP use. Self-assessment studies have shown large limitations owing to the lack of reliability in the participants' judgment. This has compelled the scientific community to develop a strategy for meaningful assessment of these new products in comparison with cigarettes. Here, we compiled a review of the existing literature on CS and halitosis and propose a 3-layer approach that combines the use of the most advanced breath analysis techniques and multi-omics analysis to define the interactions between oral bacterial species and their role in halitosis both *in vitro* and *in vivo*. Such an approach will allow us to compare the effects of different nicotine-delivery products on oral bacteria and quantify their impact on halitosis. Defining the impact of alternative nicotine-delivery products on intraoral halitosis and its associated bacteria will help the scientific community advance a step further toward understanding the safety of these products and their potentiall risks for consumers.

## Introduction

Halitosis—also known as fetor ex ore, fetor oris, bad or foul breath, breath malodor, and oral malodor—is a common condition that affects 15–60% of the population worldwide, with marked regional differences [1–3]. Halitosis is characterized by an unpleasant odor in exhaled breath [4]. The causes of halitosis are numerous and include poor oral hygiene, periodontal diseases, dry mouth, cigarette smoking (CS)/tobacco use, alcohol consumption, dietary habits, diabetes, and obesity. Other causes include aging, stress, the general hygiene of the body, and the use of certain medications [5–9].

**Figure 1:**
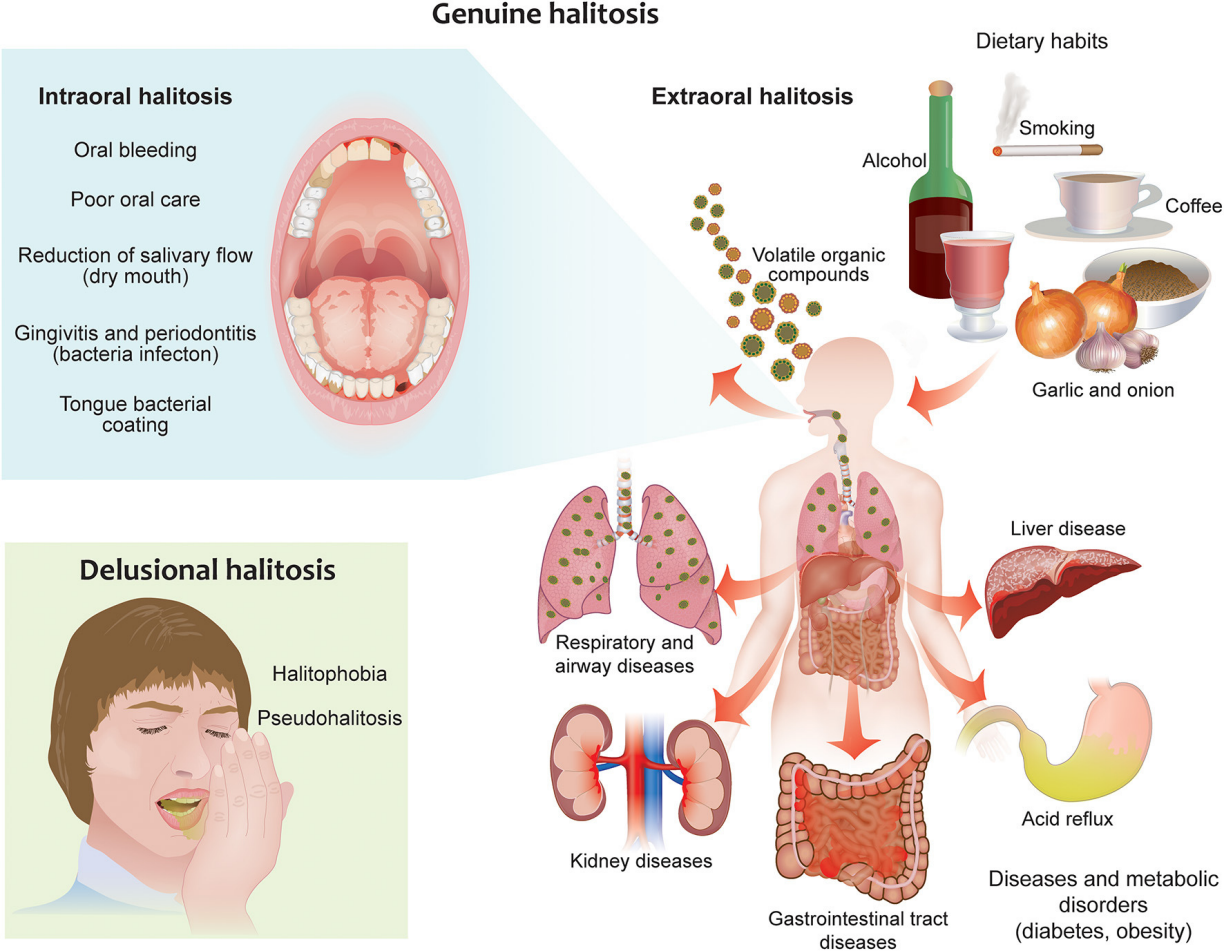
Different forms of halitosis and their causes. Genuine halitosis can be classified as intraoral or extraoral halitosis. Intraoral halitosis originates from volatile organic compounds produced by oral bacteria resulting from poor oral hygiene, dental plaque, dental caries, oral diseases, tongue coating, and dry mouth. In extraoral halitosis, the malodor is emitted from the nasal, paranasal, laryngeal, or pulmonary regions and can be caused by serious diseases like diabetes, metabolic disorders, and kidney and liver diseases. Certain foods, beverages, and lifestyle habits, like cigarette smoking and alcohol consumption, can also contribute to halitosis. Delusional halitosis occurs when no physical or social evidence exists for the presence of halitosis (pseudohalitosis); delusional halitosis may also be related to a psychogenic or psychosomatic disorder (halitophobia).

There are two types of halitosis: extraoral halitosis (EOH) and intraoral halitosis (IOH) [10]. A small percentage of halitosis cases (5–10%) are of extraoral origin. This type of halitosis can be caused by diabetes, metabolic disorders, kidney and liver diseases, as well as certain drugs and foods [11]. The vast majority of halitosis cases (80–90%), however, originate from the oral cavity; this form of halitosis is caused in general by poor oral hygiene, dental plaque, dental caries, gingivitis, stomatitis, periodontitis, tongue coating, and in rare cases oral carcinoma [12]. Dry mouth (xerostomia) might also promote oral malodor [13], although a correlation has not always been observed [14]. In healthy subjects, the most common source of oral malodor is the coating of the tongue (43.4% of cases of IOH) [15]. Tongue coating consists of bacteria, large quantities of desquamated epithelial cells, blood metabolites, food debris, and leucocytes originating from periodontal pockets, which accumulate easily because of the anatomical structure of the tongue [16]. The principal site of oral malodor is the dorsoposterior part of the tongue, which is where the most of anaerobic bacteria responsible for malodor grow [14, 16] (Figure 1).

IOH is the outcome of the production of volatile organic compounds (VOC) mainly by bacterial metabolism in the oral cavity. Almost 700 compounds have been detected in the oral cavity, of which volatile sulfuric compounds (VSC) like hydrogen sulfide and methyl mercaptan are the most abundant (90%) in IOH owing to their low odor threshold and high volatility [17–21]. Dimethyl sulfide is also a very abundant VSC in halitosis, but it is associated with EOH [22]. These VSCs are the main compounds responsible for oral malodor [23]. Other VOCs that contribute to halitosis are short-chain fatty acids, amines, and indols. VSCs are mainly produced by the metabolism of sulfur amino acids by anaerobic bacteria present in the tongue coating, and the quantity of volatiles produced is associated with the population density of the bacteria on the tongue [24]. VSCs and other VOCs have been shown to be deleterious to oral soft tissues and to induce changes that may lead to carcinogenesis [25–27]. Other compounds responsible for halitosis are diamines, such as trimethylamine, putrescine and cadaverine, produced by the putrefaction of food by bacteria [28].

The impact of cigarettes and alternative nicotine-delivery products on halitosis has not been systematically addressed so far. In this document, we will review the studies conducted on this topic and propose a framework for the systematic assessment and comparison of the effects of cigarette smoke and alternative nicotine-delivery products on IOH by leveraging state-of-the-art omics technologies for breath and microbiome analysis.

## Oral Microbiome and Halitosis

In humans, the oral cavity contains a high density and diversity of bacteria, second only to the colon [29]. It is estimated that the bacteria populating the oral cavity belong to around 700 taxa [30]. The composition of tongue microbiota is of primary importance in the development of IOH [31]. The tongue structure presents papillae, deep fissures, and crypts, which represent a niche with low oxygen potential, ideal for the growth of anaerobic bacteria associated with halitosis [32]. The tongue bacterial diversity in halitosis patients seems to be higher than that of controls [5]; it has been observed that the bacterial diversity in halitosis patients (*n* = 16; analyzed by 16S rRNA gene pyrosequencing and metagenomics methods) encompasses a wide range of microbial communities, including 13 phyla, 23 classes, 37 orders, 134 genera, 266 species, and 349 operational taxonomic units [33]. IOH is associated with the increased activity or abundance of certain bacterial genera, such as *Fusobacterium, Porphyromonas, Prevotella*, and *Tannerella*, in tongue biofilms [34, 35]. A specific biofilm on the dorsal part of the tongue seems responsible for halitosis: A study that combined fluorescence *in situ* hybridization and confocal laser microscopy analyses identified that *Fusobacterium nucleatum* and *Streptococcus* spp. cover a significant proportion of the bacterial biofilm in halitosis patients [36]. Another study applied an untargeted approach and identified that *Actinomyces graevenitzii* and *Veillonella rogosae* are closely related to the occurrence of IOH; according to this study, *Streptococcus mitis/oralis, Streptococcus pseudopneumoniae*, and *Salmonella infantis*, as well as *Prevotella* spp. are often detected in the tongue coating of halitosis patients [37]. The density of bacterial population on the tongue but not the thickness of the biofilm seems to correlate with the production of VSCs [38–40].

The prevalence of gram-negative bacteria is associated with halitosis [41]. Among gram-negative bacteria, *Prevotella, Alloprevotella, Leptotrichia*, and *Peptostreptococcus* are present at a higher percentage in subjects with halitosis than in healthy subjects [33]. Gram-negative anaerobes are the most active producers of hydrogen sulfide; among these bacteria, *Porphyromonas gingivalis, Treponema denticola*, and *Tannerella forsythia*, which belong to the red complex, are associated with periodontal diseases and positively correlated with IOH [5, 22, 42]. For example, it has been shown that hydrogen sulfide and methyl mercaptan are abundantly produced at sites of periodontal inflammation [43–45]. Moreover, *Porphyromonas* spp., *Prevotella* spp., and *Treponema denticola* may play a crucial role in providing amino acids to other anaerobic bacteria to produce hydrogen sulfide and methyl mercaptan [46]. *Porphyromonas gingivalis, Prevotella intermedia*, and *Fusobacterium nucleatum* are the major producers of indole and skatole (also called 3-methylindole) [47]. Gram-positive bacteria have also a function in IOH: They can support gram-negative anaerobic bacteria by removing sugar chains from glycoproteins and providing necessary proteins during the proteolytic processes [46].

CS has a major impact on the composition of the oral microbiome. In the next section, we will discuss the current knowledge on the connection between CS, microbiome, and IOH.

## CS, Oral Bacteria, and Halitosis

**Figure 2:**
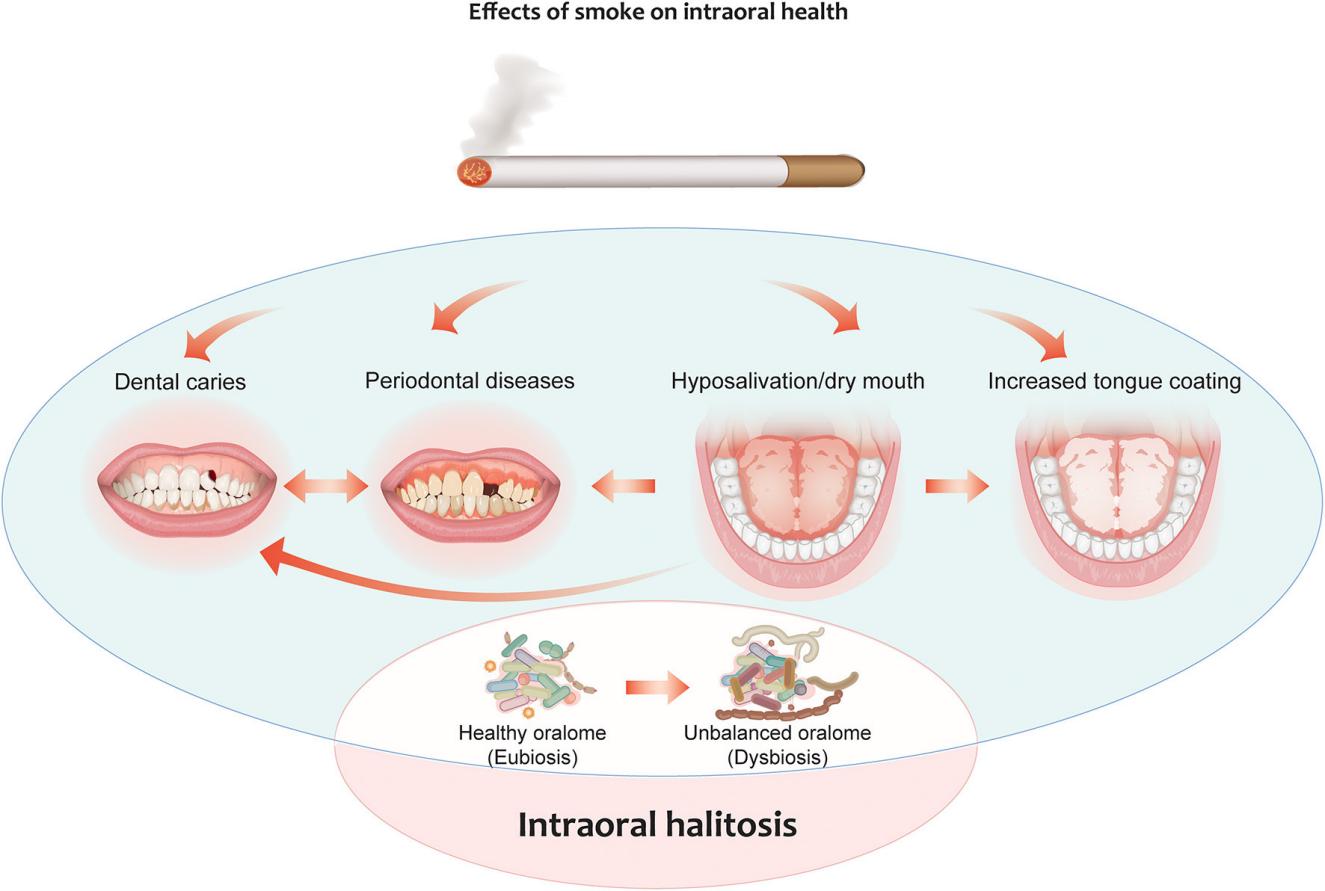
Effects of cigarette smoking on intraoral halitosis. Cigarette smoke can contribute to halitosis by causing hyposalivation, thereby facilitating the formation of deposits on the tongue. It can also contribute to the onset of periodontal diseases and the formation of dental caries. All these effects can decrease the commensal population in the oral cavity, facilitating the acquisition and colonization of periodontal pathogens. This leads to oral dysbiosis, which can, in turn, lead to intraoral halitosis.

CS is considered to be one of the major risk factors for the development of several health conditions and diseases, including many oral diseases. CS can contribute to halitosis by causing hyposalivation, thereby facilitating the formation of deposits on the tongue [48]. CS can also contribute to the onset of periodontal diseases, which are strictly correlated with alterations in the oral microbiome composition [49, 50]. Cigarette smoke contains many toxicants that can alter the oral microbiome through different mechanisms such as antibiotic effects and oxygen deprivation [51]. Lower salivary pH has been observed in smokers compared to healthy controls leading to microbial unbalance and erosion of the enamel [52]. CS can decrease the commensal population in the oral cavity, facilitating the acquisition and colonization of periodontal pathogens (Figure 2). Smokers exhibit a distinct subgingival microbial composition than non-smokers [53], although previous studies have reported conflicting results [reviewed in [50]], likely because of differences in the sensitivity and specificity of the methods used. CS has been shown to promote the cariogenic activity of oral bacteria [54]. The tongue bacterial composition of current smokers has been found to be different from that of non-smokers: A recent study by Sato et al. [55] identified the anaerobe *Veillonella dispar* as the most differentially abundant bacterial species in the tongue coating of smokers vs. non-smokers. This specific *Veillonella* species was reported to produce hydrogen sulfide as a product of L-cysteine metabolism [56]. Interestingly, *Candida* species have also been found to be some of the most abundant species in smokers with halitosis [57]. Al-Zyoud and colleagues identified a bacterial signature in the saliva of smokers that included *Prevotella, Streptococcus, and Veillonella*, which are bacterial genera that include strains associated with oral malodor; however, the authors did not perform any investigation at the species level or analyze for correlation with halitosis [58]. In another study that analyzes the bacterial composition of the saliva and tongue coating of smokers and non-smokers, the levels of *Fusobacterium nucleatum* and *Campylobacter rectus* in smokers were 5-fold greater than those in non-smokers and showed positive correlation with VSC levels [59].

Halitosis is still one of the major concerns in smokers: In a recent survey, 73% of smokers reported that they are afraid of developing such a condition [60]. Cigarette smokers are indeed reported to have a higher measured and self-perceived incidence of halitosis than non-smokers [61]. In the literature, cigarette smoke-related halitosis is also defined “smoker's breath” [14]. CS contains various components, such as acetaldehyde, benzene, and ammonia, that can confound the measurement of halitosis by organoleptic tests [62, 63]. Moreover, some problems with interobserver and intraobserver reproducibility have been described [64]. A recent study showed that smokers have a greater probability of less objectively reporting their gingival conditions and halitosis than nonsmokers [6–8]. Unfortunately, most studies that have assessed halitosis in smokers have been conducted through self-assessment surveys (Table 1). Of the five studies that combined self-reported halitosis diagnosis and halitosis measurement by organoleptic judges or by Halimeter results, four did not find a positive correlation between the self-reported halitosis and instrument/organoleptic results [70, 72, 81, 82], while the remaining one study did [85]. This difference in self-reported halitosis and instrument/organoleptic results may be due to the decreased olfactory sensitivity of smokers, which biases their self-perception of breath [1]. Therefore, the use of instruments for measuring oral malodor combined with organoleptic judgement may provide a more accurate diagnosis. To date, only few studies have systematically addressed the correlation of CS with halitosis. One study in subjects with periodontitis found that VSC levels were higher in the gingival pockets of smokers than in non-smokers [86]. In other studies, VSCs and organoleptic scores were significantly associated with bacterial tongue coating and CS [81, 82]. Another study showed that smoking was associated with self-reported halitosis but not with VSC levels [72]. It is noteworthy that some studies have found that smokers have poorer oral hygiene than non-smokers, and this also could play a role in the development of IOH [71, 87].

**Table 1:** Studies that have investigated the impact of cigarette smoking on halitosis.

**Reference**	**Total study participants**	**Smoking status**	**Diagnosis of halitosis**	**Conclusions**
Al Ansari et al. [65]	1,551 (dentistry patients and random subjects)	Current smokers/former smokers	Self-reported	Halitosis perception significantly higher in smokers and former smokers than in non-smokers
Alqahtani et al. [66]	100 (peri-implantitis patients)	Current smokers/waterpipe users	Self-reported	Significantly more smokers and waterpipe users reported halitosis than non-smokers
AlSadhan [3]	2,343 (random subjects from a school and government office)	Current smokers/waterpipe users	Self-reported	Smoking and waterpipe use was significantly associated with halitosis
Al-Zahrani et al. [67]	38 (diabetes patients)	Current smokers/former smokers	Self-reported	No significant association between smoking and halitosis, although the study contained only one current smoker
Ayo-Yusuf et al. [68]	896 (dentistry patients)	Current smokers	Organoleptic measurement	Smoking significantly associated with high organoleptic ratings
Babazadeh et al. [69]	519 (adolescents)	Current smokers/waterpipe users	Self-reported	Halitosis associated with poor oral health in smokers
Eldarrat et al. [70]	233 (undergraduate and graduate students)	Current smokers	Self-reported	Smoking was not correlated with halitosis, although a greater number of smokers reported halitosis than non-smokers
Jiun et al. [71]	200 (dentistry patients)	Current smokers	Halimeter	75% of smoking subjects show halitosis vs. 8% of non-smokers; the difference was statistically significant
Lee et al. [72]	54 (visitors at a health center)	Current smokers/former smokers	Self-reported, Halimeter	Smoking significantly associated with self-conscious and self-reported halitosis but not associated with halitosis diagnosed by a Halimeter
Rech et al. [73]	48 (pneumology clinic patients and random subjects)	Current smokers	Self-reported	33% of smokers reported halitosis vs. 4.2% of non-smokers. Halitosis more common in subjects who had smoked for more than 20 years
Romano et al. [74]	736 (Dentistry patients)	Current smokers	Self-reported, organoleptic measurement	Heavy smoking negatively associated with the concordance of self-reporting and organoleptic measurement of halitosis
Saadaldina et al. [75]	460 (Dentistry patients)	Current smokers	Self-reported	Smoking significantly associated with halitosis
Şanli et al. [76]	1,840 (ear, nose, and throat clinic patients)	Current smokers/former smokers	Self-reported	37.7% of smokers reported halitosis vs. 22.4% of non-smokers
Struch et al. [77]	3,005 (random subjects [citizens])	Current smokers/former smokers	Self-reported	Former smokers and smokers reported halitosis more often than never smokers
Setia et al. [78]	277 (dental students)	Current smokers	Self-reported	Halitosis reported by 80% of smokers
Silva et al. [45]	900 (random subjects [citizens])	Current smokers	Self-reported	Interaction of the effects of smoking and periodontitis on halitosis
Tubaishat et al. [79]	580 (random subjects from a school)	Current smokers	Self-reported	58.5% of smokers reported halitosis
Barik et al. [80]	16,354 (random subjects [citizens])	Current smokers/smokeless tobacco users	Self-reported	0.2% of smokers and 0.1% of smokeless tobacco users reported halitosis
Bornstein et al. [81]	419 (random subjects [citizens])	Current smokers	Self-reported, organoleptic measurement, Halimeter	Positive correlation of smoking, tongue coating, and periodontal screening index with halitosis; weak correlation between self-reported halitosis and VSC measurements and organoleptic scores
Bornstein et al. [82]	626 (army recruits)	Current smokers	Self-reported, organoleptic measurement, Halimeter	Positive correlation of smoking and tongue coating with halitosis; inverse correlation between smoking and VSC levels; no correlation between self-reported halitosis and organoleptic scores or VSC measurements
Kayombo et al. [83]	400 (workers)	Current smokers	Self-reported	25.8% of smokers reported halitosis; the association was statistically significant
Kim et al. [84]	359,263 (adolescents)	Current smokers	Self-reported	Smoking not statistically correlated with halitosis; smokers' cohort was small
Miyazaki et al. [85]	2,672 (workers)	Current smokers	Self-reported, Halimeter	Smoking, tongue coating, and self-diagnosis significantly associated with VSC production
Khaira et al. [86]	33 (periodontitis patients)	Current smokers	Perio2000 system	Higher percentage of sites with VSCs in smokers than in non-smokers

*Data accessed in June 2021*.

Quitting smoking has been shown to shift the composition of the oral bacterial population in healthy subjects to a composition similar to that in never smokers, indicating that the cigarette smoke-induced changes in the microbiome are reversible [88]. However, no information has been published on the correlation of quitting CS with halitosis improvement. Interestingly, reduced risk of halitosis is among the suggested reasons for quitting smoking by dentist guidelines in the United Kingdom [89].

## Impact of Alternative Nicotine-Delivery Products on Halitosis

While cessation is the best practice for reducing the negative effects of CS, only a small percentage of smokers who attempt to quit do not relapse 6 months after [90]. The tobacco harm reduction strategy has recently emerged as a complementary approach for helping reduce the adverse effects of CS [91]. The concept of tobacco harm reduction is to switch smokers to less harmful products that emit significantly lower levels of toxicants while still delivering nicotine at comparable levels to cigarettes [92, 93]. Currently, two classes of products that have the potential to fulfill these characteristics are being marketed: electronic vapor products (EVP) and heated tobacco products (HTP). It is important to conduct robust scientific studies to determine the reduced risk potential of these products in comparison with cigarette smoke.

So far, very few studies have investigated halitosis in relation to such alternative products and CS. EVPs and HTPs are generally perceived to have no impact on breath or less impact than cigarette smoke. A recent automated data mining study investigated more than 41,000 online forum posts related to the effects of EVPs [94] and identified only 5 posts in relation to halitosis and EVPs. Another survey investigated HTPs and EVPs and found that only 9 and 6% of users, respectively, reported halitosis vs. 86% of cigarette smokers [61]. In a study by Soule et al., a cluster of 12 patients out of 49 EVP users reported a bad taste in the mouth, deadened taste buds/sense of taste, bad breath, or a metallic taste in the mouth [95]; the authors did not report the actual frequency of bad breath (halitosis).

However, as previously mentioned, self-reported halitosis often does not correspond to objective (measured) halitosis [81, 82, 96]. A recent microbiome study suggested that the salivary bacterial composition of EVP users with periodontitis varies from that of never smokers and smokers and shows more similarities with the composition in non-smokers than smokers [97]. Another recent study investigated the microbiome profile in saliva and buccal samples of EVP users compared with non-smokers/non-vapers, finding significant changes in the diversity composition in the saliva between the 2 groups [98]. However, these studies did not investigate the possible correlation between a shift in the bacterial composition and halitosis. The use of electronic nicotine-delivery products is speculated to lead to bad breath due to the dry mouth (xerostomia) attributed to the nicotine-related inhibition of salivary flow [99]. Xerostomia can also lead to an imbalance in the oral bacterial population, tipping it toward a greater prevalence of anaerobic species, which are often related with halitosis [8]. However, no study has performed a systematic measurement of halitosis-related compounds in EVP or HTP users so far.

## Assessment of Halitosis

Halitosis is a source of embarrassment and social challenge, and it significantly influences the social life of affected subjects [100]. A study conducted in the Netherlands showed that halitosis was among the 100 most common causes of distress in the human population [101]. Many people who experience halitosis seek medical advice, and halitosis can be the third most common reason for dentistry visits, behind dental caries and periodontitis [102]. The social pressure of having a fresh breath pushes many people to be preoccupied about this condition. However, self-perceived oral malodor does not always reflect a clinical condition, and it is, therefore, important to discriminate between “genuine halitosis,” when the malodor is easily recognizable, and “delusional halitosis,” which may instead relate to a psychogenic or psychosomatic disorder (halitophobia) [103]. Delusional halitosis may indicate depression or obsessive–compulsive behavior, necessitating psychiatric care [101]. Anxiety itself increases the oral levels of malodor-associated compounds; consequently, many professionals do not consider self-reporting of halitosis reliable [104]. Assessment methods are, therefore, important for discriminating genuine halitosis from delusional halitosis and for determining the severity of the condition.

In the context of tobacco research, systematic measurement of the volatile compounds present in the breath of EVP and HTP users will help understand the potentially reduced impact of these products on breath odor in comparison with cigarette smoke.

For an exhaled breath analysis, some general rules regarding beverage consumption and personal hygiene should be followed to minimize any interference that may cause results to be interpreted incorrectly. The general instructions for participants are to avoid using a mouthwash for at least 30 min prior to the exhalation measurement, refrain from eating, smoking, drinking (besides water), or any oral hygiene activity for 1–2 h prior to the test [105]. In addition, participants should avoid consuming garlic, onions, or spicy food for 2 days before their appointment (as these foods are a source of sulfur compounds) and also refrain from drinking alcohol or coffee for 12 h before the measurement [21]. The potential of certain foods and beverages to mask odor need to be considered. Hansanugrum and Barringer reported the benefits of drinking whole milk as a way to reduce the presence of diallyl disulfide and allyl methyl disulfide after garlic consumption [106]. Similarly, the levels of methanethiols and allylthiols are significantly reduced when garlic is rinsed with a mushroom (*Agaricus bisporus*) extract before ingestion [107]. Enzymatic deodorization involving oxidation of polyphenolic compounds by enzymes (e.g., alliinase) as well as the presence of polyphenols without enzyme activity or acidic deodorization have been proposed as tools for reducing oral malodor [108, 109].

Diagnostic methods rely primarily on organoleptic (intensity) or hedonic (pleasant/unpleasant) assessment of breath by trained panel. This approach is considered the standard for halitosis diagnosis [110]. However, it can be difficult to compare results across different trained sensory panelists, as the methodology may vary in terms of both the definition of odor attributes as well as the intensity ranking scale. Analytical technique gas chromatography (GC) coupled to mass spectromety (MS), considered as the gold standard for VOC analysis, has been applied to identify chemical markers associated with malodor. The findings of studies employing these methods have led to the development of different sensor (portable) instruments for halitosis diagnosis (Table 2) [111]. As an example, total sulfuric compound measurements can be monitored by using a portable Halimeter (Interscan Corporation, Chatsworth, CA, USA), which is based on a volumetric non-selective gas sensor. This instrument has been shown to provide good reproducibility. However, the main drawback of the Halimeter is that it cannot discriminate different VSCs [110]. By contrast, OralChroma (Abimedical, Abilit Corp., Osaka, Japan) can differentiate between hydrogen sulfide, methyl mercaptan, and dimethyl sulfide by means of rapid GC separation [110].

**Table 2:** Comparison of direct exhaled breath measurement methods applicable in halitosis research.

**Direct measurement methods applicable in halitosis research**	**Implemented in halitosis research**	**Sensitivity level of detection**	**Quantification**	**Untargeted analysis (biomarker research)**
Organoleptic	YES	Only approach that can predict the degree of odor that a gas mixture may impart to the human nose	NO Exhaled breath smell is ranked from 0 (undetectable) to 5 (heavy foul odor)	NO
**Sensors**				
Halimeter (Interscan Corporation)	YES	ppb	YES • Provides quantification of total sulfuric compounds, • Cannot differentiate between sulfuric compounds, • Insensitive to non-sulfuric volatile compounds	NO
Oralchroma (Abimedical)	YES	ppb	YES • Quantification of H_2_S, CH_3_SH, (CH_3_)_2_S, • Insensitive to non-sulfuric volatile compounds	NO
NeOse (Aryballe)	YES	Compound-dependent Ammonia (LOD 20 ppb) Hydrogen sulfide (LOD 50 ppb)	NO • “Digital olfaction”, • Can identify different odors on the basis of records within an AI database	NO
**Real-time Ms techniques**				
SIFT-MS	YES	ppt (Compound-dependent)	YES	NO (More targeted approach)
PTR-MS	NO	ppt (Compound-dependent)	YES	YES (Low capability for compound identification)
Super SESI-HR-MS	NO	ppt (Compound-dependent)	NO	YES (Enhanced capabilities for compound identification)

*HR, high resolution; LOD, limit of detection; MS, mass spectrometry; ppb, parts per billion; ppt, parts per trillion; PTR, proton transfer reaction; SESI, secondary electrospray ionization; SIFT, selected ion flow tube*.

Recent developments in the analysis of odors and fragrances include the introduction of the portable NeOse device (Aryballe Technologies, Grenoble, France), which is based on the silicon photonics technology [112]. Through a combination of artificial intelligence data processing, this biosensor is able to monitor VOCs and has the potential to reveal the distinct signature of halitosis [113]. A recent comparison of current measurement instruments showed that the data generated from the NeOse biosensor correlate well with both selected-ion flow-tube (SIFT) coupled to MS measurements and organoleptic scoring. This is a promising insight for the future development of point-of-care halitosis measurement methods [113].

However, these portable instruments do not provide a holistic analysis of compounds (untargeted analysis) and do not allow the discovery of novel chemical markers in exhaled breath.

Several indirect measurement techniques exist which address the presence of bacteria associated with IOH rather than the target compounds [reviewed in [114]].

### A Multiomics Approach for Holistic Understanding of Halitosis Related to the Oral Microbiome

A holistic approach can help highlight the interrelationships among oral bacterial species, their metabolism, and the volatile compounds that are ultimately responsible for halitosis. The advent of high-throughput omics technologies has allowed the detection of a wider range of bacterial taxa associated with IOH as well as the characterization of their metabolites. Combining multiple omics technologies has an advantage over using a single omics approach in that it provides a greater understanding of the disease, from its original cause (genetic, developmental, or environmental) to its functional consequences or relevant interactions [115, 116]. Integration of different omics approaches may help us gather comprehensive information on the function of the oral microbiome in halitosis [117]. The different omics data that are found to correlate with halitosis, such as microbiomics and metabolomics data, can be fit into a logical framework to discover the responsible molecular pathways that elucidate the role of the different bacteria responsible for halitosis [118, 119].

A multiomics approach may also be of great help in linking environmental factors, such as CS or the use of alternative nicotine-delivery products, to changes in the microbiome and metabolome.

#### Microbiomics

In the past, microbiome investigations have often focused on culturing techniques, which are laborious and limited to taxa that are amenable to *in vitro* culturing. In recent times, there has been a shift toward high-throughput techniques for obtaining snapshots of the composition of the microbiome. One of the main classes of molecules used for this purpose are the nucleic acids. In principle, detecting sequences distinct to a specific taxon allows us to infer the presence and even abundance of that taxon.

PCR-based methods can help identify target species in samples and have been used for investigating the link between the microbiome and halitosis [120, 121]. In fact, primer panels have been devised for oral microbiome investigations [122]. The method is generally limited to microorganisms for which sequence information is available, and it only allows a limited set of predefined organisms to be surveyed. Terminal restriction fragment length polymorphism analysis of 16S sequences has been used for species identification [40], especially in conjunction with machine learning [123], but the method has not gained popularity. The most used method currently is 16S rRNA gene sequencing, which involves amplifying and sequencing one or more of the hypervariable regions in the 16S ribosomal subunit gene and assigning a taxonomic classification on the basis of homology to known sequences. This method has been used abundantly in halitosis research [31, 33, 124] and notably in direct connection with the effect of CS on halitosis [58]. Amplicon sequencing offers an affordable undirected approach for investigating the microbiome which is not limited to specific bacteria. It is, however, limited to taxonomic profiling and cannot directly provide information on metabolic activity.

More recently, shotgun sequencing has been used for microbiome analysis. This method yields a snapshot of all the DNA in a sample. Sequencing reads can then be either assigned a provenance by homology to known microbial sequences, or assembled into “metagenome” fragments and then used for functional analysis, thus potentially obtaining information on both taxonomy and metabolism. While it is more resource-intensive than simple amplicon sequencing, shotgun sequencing might be preferable because of its broader scope of use. Large-scale metagenomics projects have already characterized several niches of the human microbiome, including the oral microbiome [125]. Other shotgun approaches have investigated the tongue microbiome of smokers vs. non-smokers [55].

#### Metabolomics

Metabolomics is a fast-emerging discipline in systems biology and represents the study of all low-molecular-weight molecules present in a biological sample. The metabolome is the sum of all molecules in a biological organism or system and usually contains vast information about the end products of cellular processes [126]. Volatilomics, breathomics, and salivaomics are three branches of metabolomics that can be applied as the most modern approaches for studying halitosis.

Volatilomics is focused on the analysis of the VOCs emitted by a living organism and is integrated with breathomics and salivaomics research [127, 128]. VOCs can be detected in the headspace area of saliva [129] and directly in exhaled breath [130]. It is mostly GC–MS detection that is used for studying the VOCs present in saliva. This technique has already been applied in halitosis research, where a study recently identified two potential biomarkers of halitosis: 5-aminovaleric acid and *N*-acetylornithine [131]. Monedeiro et al. have reported 164 VOCs in saliva, which they monitored by headspace solid-phase microextraction coupled to GC–MS (HS-SPME-GC–MS). Of the 164 VOCs, 23 are specific to halitosis (including a large number of sulfuric compounds) and 41 to abscesses (a greater variety of alcohols, aldehydes, and hydrocarbons, which are biomarkers of inflammatory processes) [132]. Another study has reported significantly higher concentrations of salivary cortisol in subjects with psychosomatic halitosis than in subjects with genuine halitosis and control subjects (*p* < 0.05) [133]. Additionally, salivary cysteine—a direct precursor of hydrogen sulfide—can be considered a reliable marker for assessing the severity of oral tissue damage in periodontitis patients [134].

Breathomics is a branch of metabolomics that explores the compounds present in human breath by using various analytical techniques [135–137]. This approach helps clarify the link between breath molecules and certain diseases/conditions [135, 138]. The list of diseases/conditions that are related to breath molecules is not limited to halitosis but also includes metabolic, lung, and gastrointestinal disorders [139]. The recently introduced human breathomics database (HBDB; https://hbdb.cmdm.tw/) provides scientists a tool for identifying and further investigating potential breath biomarkers [136]. The database is currently updated with 913 compounds that are related to 60 diseases (accessed in August 2021).

Exhaled breath analysis can involve two approaches: off-line and on-line (real-time) measurement. The off-line approach involves collection of exhaled breath, sample analysis, and data analysis [140]. Exhaled breath collection includes collection of the gas phase of exhaled breath by using sampling bags or bottles (for example) and collection of exhaled breath condensate by using a condenser. GC–MS and/or liquid chromatography coupled to MS (LC–MS) are usually used for analysis of such samples [127]. The GC–MS off-line analysis is usually performed by thermal desorption of a Tenax tube in which VOCs from the collected samples are trapped. This technique has been used by van den Velde et al. for studying VOCs associated with halitosis in non-halitosis subjects. During sample collection, the authors made a distinction between alveolar and mouth air. They instructed the subjects not to breathe for 30 s, so that they could collect mouth air by using a manual piston and Teflon bulb. They then used a commercially available Bio-VOC sampler to collect exhaled alveolar air. The authors detected 14 compounds associated with bad breath and highlighted the importance of differentiating between alveolar and mouth air because of their different chemical compositions [141].

Over the last three decades, real-time exhaled breath analysis has been used as a complementary technique to the off-line approach [142–147]. The main difference between the off-line and real-time analyses is that the latter provides (i) results quickly and (ii) does not include exhaled breath sample collection and storage, which can cause loss and degradation of compounds. Bruderer et al. have recently reviewed the various methods that allow real-time analysis of exhaled breath [148], including the three main approaches: proton transfer reaction (PTR-MS], selected-ion flow-tube (SIFT-MS), and the more recent secondary electrospray ionization (Super SESI) MS (Table 2). These approaches allow measurement of oral malodor directly in the oral cavity and can also be adapted for measurement of bacteria cultivated *in vitro* [149].

SIFT-MS has already been employed as a real-time analytical method for monitoring and quantifying hydrogen sulfide, methanethiol, and dimethyl sulfide in research related to mitigating the effects of a dentifrice on halitosis [23]. SIFT-MS has proven to be the technique of choice for detecting and quantifying VSCs in exhaled air with high sensitivity up to the low ppb levels. It is perfectly suited for targeted analysis and can provide accurate concentration measurements. However, the main drawback of this technique is that it currently cannot be applied in untargeted analysis (which would allow discovery of new biomarkers), as the mass analyzer does not offer high-resolution accurate mass analysis capabilities [148] (Table 2).

To our knowledge, neither PTR-MS nor Super SESI-MS has yet been used in halitosis research. PTR-MS can be successfully applied for targeted analysis of the VOCs present in exhaled air, and it can provide quantification up to ppt levels [150]. The exhaled breath VOC levels measured by PTR-MS can be affected by variations in humidity and CO_2_ and O_2_ levels [139]. The main drawback of this technique is its low-resolution mass accuracy capability (most PTR time-of-flight mass analyzers have a resolving power [m/Δm] of >6,000) [151]. Similar to SIFT-MS, PTR-MS is not suited for untargeted identification of chemical markers (Table 2).

Super SESI-HR-MS is the latest among these real-time techniques. It has the main advantages of being able to operate at ambient pressure and couple with any ambient inlet MS, including high-resolution accurate mass capability when coupled to an Orbitrap mass analyzer (m/Δm up to 240,000 with a Q Exactive HF series). Moreover, this instrument can generate high-resolution accurate mass MS/MS spectra, which are particularly useful for distinguishing between compounds that have similar elemental formulas [148], hence allowing detection and identification of new biomarkers related to halitosis. The current main drawback of Super SESI-HR-MS compared with SIFT-MS and PTR-MS is that it does not provide absolute quantification [148] (Table 2). All real-time approaches have the disadvantages of high cost and lack of suitability outside a laboratory setting.

#### Integrative Analysis

Often, studies that employ high-throughput methods (i.e., omics) consider only one technique. Using multiple techniques in parallel can yield information that is not only mutually supportive but may also be combined into a synergistic model that could be greater than the sum of its parts. There are numerous *in vivo* interactions between microbial organisms, with both inter- and intra-specific mechanisms that can influence microbial growth or activity. These include crossfeeding between species, biofilm production, quorum sensing, and interspecific competition or “arms race.”

Some of these interactions and mechanisms have been characterized for oral diseases such as periodontitis and could potentially be harnessed for understanding and limiting halitosis. For example, crossfeeding microbial species rely on other species for their growth and even survival [152]. Additionally, crossfeeding in the oral cavity is necessary for some pathogens at certain points in their lifecycle. Identifying such relationships among pathogenic or halitotic species might help us better understand the pathogenicity of these dependent species and allow us to target upstream metabolite suppliers for intervention.

Conversely, there is also evidence of repression networks, whereby commensals are found to repress the growth of pathogens [152]. For example, there is *in vitro* evidence of interspecies competition suppressing a pathogen, although its *in vivo* applicability remains unknown [153]. Furthermore, there are known effector proteins which can inhibit the development of pathogenic bacteria [154]. If a relationship can be established between the absence of commensals and halitosis, it would open new avenues of treatment, including supplementation of missing commensals in treatment products. Furthermore, quorum-sensing molecules, which can influence pathogen activity, may offer a point of intervention for suppressing pathogen growth and invasiveness [155]. Such molecules have been suggested as a method of preventing other biofilm-related diseases in the oral cavity [156].

Using an untargeted metabolomics approach, Seerangaiyan et al. have recently identified 39 metabolites that are putatively associated with IOH in tongue scrapes from patients [117]. The authors also proposed a list of bacterial classes that might be associated with these metabolites. *In vitro* experiments on key bacterial species associated with halitosis may also provide an initial understanding of the malodorous compounds that these species produce and eventually the pathways that may be involved in this process [38]. Less-known malodorous compounds than hydrogen sulfide and methyl mercaptan—such as short-chain fatty acids, polyamines, and indole—do play a role in halitosis, although not much knowledge exists on their association with bacteria [157]. Further identification of the bacteria responsible for producing these compounds may help guide new therapeutic strategies. Some authors have reported the benefit of using Annotation of Metabolite Origins via Networks (AMON] [158] as an obvious route for identifying the origin of metabolites through genomic information and for visualizing potential host–microbe interplays when integrating microbiome and metabolome datasets. Mechanistic relationships between microbial communities and host phenotypes can be better understood through integrated analysis of microbiome and metabolome data.

## A Framework for Studying Halitosis in the Context of CS and Alternative Tobacco Product Use

**Figure 3:**
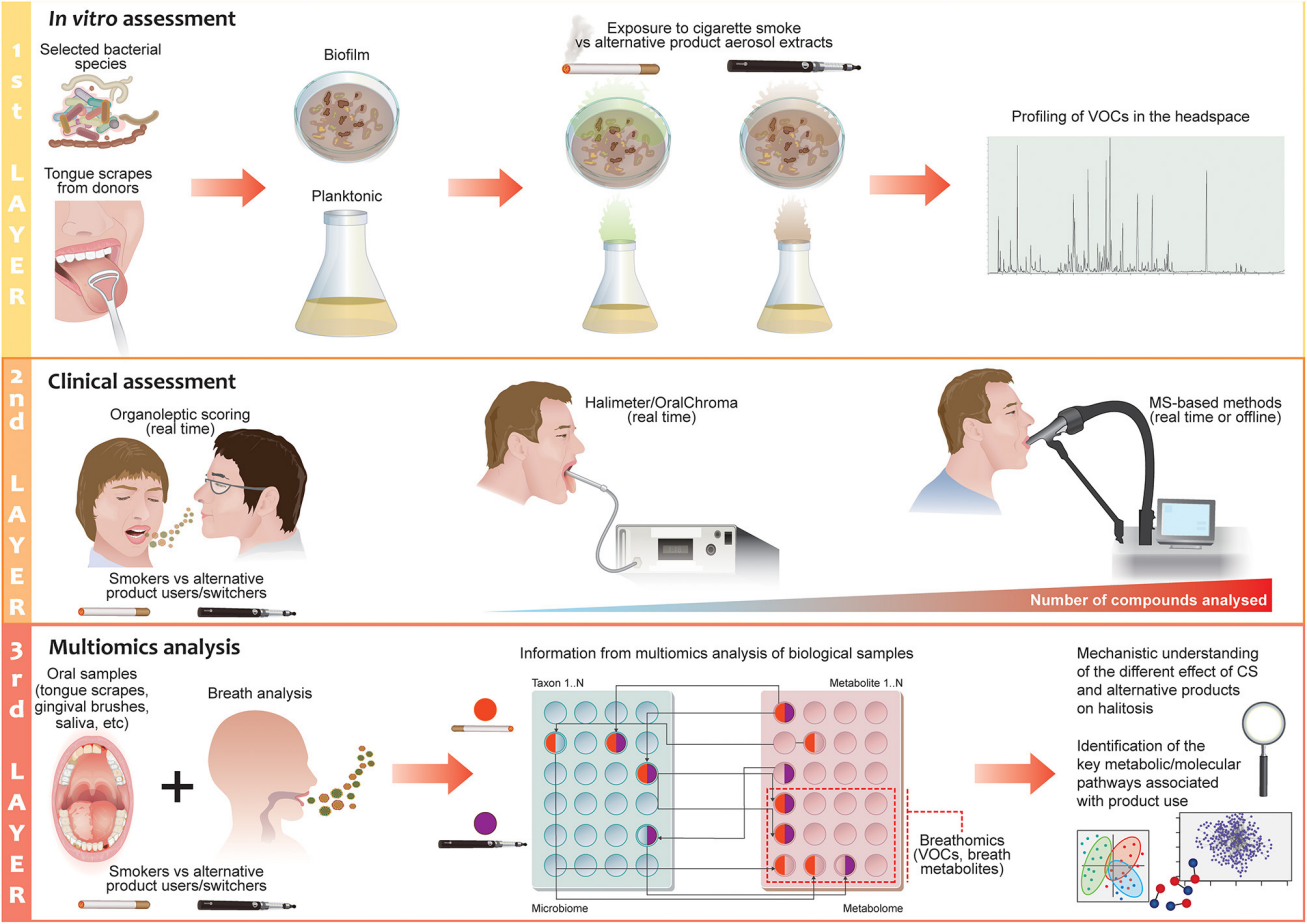
The 3-layer halitosis analysis framework for cigarette smoking and alternative nicotine-delivery product use. The first layer of the proposed framework focuses on testing the effects of the aerosols or liquid fractions of alternative nicotine-delivery products in comparison to cigarette smoke/extract *in vitro*. Selected bacterial species with known effects on halitosis or tongue scrapes from halitosis patients/donors can be employed in planktonic or biofilm models; the headspace of the flasks or plates is analyzed to detect volatile organic compounds by using MS-based instruments. The second layer focuses on validating the results from the *in vitro*/*ex vivo* experiments with clinical measurements of the breath of cigarette smokers and users who have switched to alternative nicotine-delivery products. Real-time breath measurements can be performed with MS-based techniques in a laboratory setting to discover new potential biomarkers of halitosis and provide quantitative measurement of a halitosis-related compound. Evaluation by organoleptic judges is incorporated in the design of the clinical study to define the degree of odor in the subjects. The third layer of the investigation focuses on gaining a mechanistic understanding of the causes of halitosis in relation to CS or alternative nicotine-delivery product use by applying a multiomics approach that encompassed microbiomics and metabolomics.

From the body of scientific publications available on halitosis in relation to CS, it is evident that most results were produced from self-assessment evaluations. In general, when a study employed organoleptic assessment and instrument analysis, the results did not correspond with the self-assessment results. Moreover, self-assessment does not provide information on the origin of halitosis, its intensity, or the type of compounds/bacteria that may be responsible for this condition. In the context of comparing the effects of different tobacco/nicotine-containing products on halitosis, it is necessary to have a structured approach that allows us to obtain quantitative results from which we can glean mechanistic insights. In particular, according to the tobacco harm reduction strategy, comparison of alternative nicotine-delivery products with cigarettes is of paramount importance for demonstrating a potential risk reduction [92, 93]. Any approach for studying these products should, therefore, answer the following questions: (1) What is the impact of alternative nicotine-delivery products on halitosis relative to cigarette smoke; (2) how does the oral bacterial composition differ between cigarette smokers and alternative nicotine-delivery product users in relation to halitosis; and (3) how does the bacterial composition influence the production of halitosis-related compounds? To answer these questions, we should consider a multilayer framework assessment (Figure 3).

The first layer of the assessment should aim to quickly and cost-effectively prescreen the effects of different product extracts on selected bacterial species with known effects on halitosis in an *in vitro* system. Such a platform would employ planktonic models or biofilm models, derived from either a single bacterial species, a synthetic community of bacteria, or a natural community of bacteria from tongue scrapings or gingival brushes from different donors. Donors with different grades of halitosis could also be employed, as these are more representative of the halitotic oral microbiome. The types of analyses that can be performed have been demonstrated by Chatzigiannidou et al. who used both synthetic communities and *ex vivo* samples to shed light on the changes in microbiome composition and viability in response to chlorhexidine [159]. Such methods could be extended to investigate other compounds, as well as investigating the effects of cigarette smoke on bacteria or bacterial communities. Other examples of the use of this platform can be found in the Saad and co-workers publications: they cultivated the biofilm from tongue scrapes of halitosis patients to test efficiently toothpaste and mouthwash formulations *in vitro* [160, 161].

Detection and identification of volatile compounds should be performed in the headspace of the flasks or plates by using MS-based instruments (SIFT-MS, super SESI-HR-MS, HS-SPME-GC–MS) that are preferably directly connected to the system [149, 160, 162, 163]. A perfusion matrix flow system can maintain a tongue-derived microenvironment which can be conveniently measured or monitored, so that it can be used for studying VOC/VSC production. Such a dynamic system can be maintained for several days, possibly weeks, allowing similarity to *in vivo* conditions [160]. This model can be used to replicate many of the essential biotic and abiotic features of a real oral biofilm, including ecological stability, and the collective microbial activity that results in VSC production following exposure to different tobacco/nicotine-delivery products, which can be administered at this stage in the form of liquid extracts or aerosols. Nicotine should be used as the reference compound for comparing the products: Cigarette smokers have been shown to adjust their puffing pattern to obtain a certain nicotine concentration in the blood, and the same has been shown in EVP users [164]. This suggests that smokers who switch to an alternative nicotine-delivery product will maintain the same nicotine intake that they had when smoking cigarettes.

The second layer of assessment should focus on exhaled breath analysis. The breath of cigarette smokers and users who have switched to alternative nicotine-delivery products would be analyzed; smokers that quit should be also employed as control group. Real-time breath measurements (Super SESI-HR-MS) can be performed to discover new potential biomarkers of halitosis and to obtain a quantitative measurement of halitosis-related compounds (SIFT-MS or PTR-MS). In addition, off-line GC–MS can be applied as a complementary technique for qualitative and quantitative analysis. Although these measurements can provide a large quantity of data on compounds related to halitosis, they cannot yet predict the degree of odor that a gas mixture may impart to the human nose. Therefore, it is fundamental that evaluation by organoleptic judges is incorporated in the design of a clinical study [113].

The third layer of the investigation should focus on gaining a mechanistic understanding of the causes of halitosis in relation to CS or alternative nicotine-delivery product use. As the bacterial component plays a major role in IOH, as described previously, bacterial samples could be sampled from the tongue surface, supra and sub-gingival plaques, saliva, swabs of oral and nasal cavities, etc.; a multiomics approach that encompasses microbiomics, metabolomics, and proteomics may provide a unique opportunity to define bacterial interactions and detect species-specific metabolite profiles. Finally, this analysis will provide a mechanistic understanding of the different effects of cigarette smoke and alternative nicotine-delivery products on halitosis and allow us to identify the key metabolic/molecular pathways associated with the use of such products. A correlation of these results with the *in vitro* results from the first layer would be useful to optimize the *in vitro* methods of bacterial biofilm for research applications.

## Clinical Applicability of the Framework and Perspectives

The hypotheses derived from the experimental work described above could be further validated in a clinical setting where subjects are confined to measure halitosis changes. We propose a randomized, controlled, open-label, three-arm parallel group study to evaluate the effect of switching to alternative nicotine-delivery products in healthy smokers suffering from halitosis. The three study arms would comprise a group of healthy smokers who continue to smoke, a group of healthy smokers who switch to alternative nicotine-delivery products, and a group of healthy smokers who quit smoking. The quitting group would be the control, to understand whether the changes in the halitosis profile of the switchers are more similar to the effects of quitting smoking rather than to continuing CS. It may be necessary to conduct a pilot study to estimate the effect size and the required duration for the main study.

The advantage of confinement is that it would allow control over confounding factors that might enhance or mask the detection of halitosis, such as level of hydration, food and beverage consumption, quantity of cigarettes or other products used (see section Assessment of Halitosis). Subjects suffering from caries, periodontitis or gingivitis should be excluded from the study because their condition would make the dominant contribution to halitosis, potentially masking the effects of smoking or switching. Additional information regarding oral and overall health, including age and gender, diet, and the use of antibiotics or other medications could be used to stratify and match subjects among the groups. Organoleptic judges could be employed to score oral malodor at baseline and at the completion of the study.

Devices sensing the presence of VOCs or VSCs (such as the Halimeter) could facilitate the diagnosis of halitosis in addition to traditional organoleptic measurements. MS-based methods could be employed to characterize the VOC and VSC profiles in the breath of the subjects. During the study, microbiome samples could be collected from locations within the oral cavity: tongue surface, supra- and subgingival plaques, saliva, and swabs of oral and nasal cavities. Characterization of different microbial population of different oral locations and volatile compounds detected in exhaled breath (layer 3 of the framework described above) would contribute to understanding of changes in oral bacterial populations upon switching from smoking to an alternative nicotine-delivery product.

The findings might provide a better understanding of the causes of halitosis and allow identification of specific biomarkers in oral bacterial/VOC composition that could be targeted in the development of halitosis treatments, not only in smokers and switchers to alternative nicotine-delivery products, but also in all halitosis patients. These findings might ultimately enable the development of personalized solutions based on an individual's microbial and VOC profile.

Although the proposed framework is described in the specific context of the study of IOH produced by oral bacteria, layers 2 and 3 would also be applicable to EOH patients. Besides the interpretation of metabolomics results in relation to the oral microbial population, the metabolic profile of the exhaled breath could also provide important insights into EOH in patients with known diseases or conditions and lead to the identification of novel biomarkers [110]. In this way, if a metabolic signature in the breath could be related consistently to a certain disease or condition, MS-based methods or sensors could be employed in diagnosis. For example, once validated, portable devices such as NeOse could be used by dentists in daily practice [110] and may become a useful tool to support the organoleptic scoring by judges and ensure inter-study reproducibility of their assessments.

## Conclusions

CS is a major risk factor for halitosis. For smokers who are not able to quit, alternative nicotine-delivery products like EVPs and HTPs may help reduce the health risks associated with CS. To date, only a few systematic studies have analyzed the effects of CS on halitosis, and none has assessed the effects of EVPs and HTPs on this condition. Self-assessment studies have shown huge limitations owing to the lack of reliability in the participants' judgment. This compels the scientific community to develop a strategy for meaningful assessment of these new products in comparison with cigarettes. In this review, we proposed a 3-layer approach that combines the use of the most advanced breath analysis techniques and multiomics analysis to define the interactions between oral bacterial species and their role in halitosis *in vitro* and *in vivo*. Such an approach will allow us to compare the effects of different nicotine-delivery products on oral bacteria and quantify their impact on halitosis. Our proposed framework has the potential to quantify and mechanistically address the impact of alternative nicotine-delivery product use in comparison with cigarette smoke on halitosis. The results from such a comprehensive analysis could be used to design treatments for mitigating the potential side effects of alternative nicotine-delivery products on breath odor. Finally, the proposed framework will be an important step for further defining the safety and risks associated with the use of these products for consumers.

## Author Contributions

FZ: conceptualization and writing. TZ, JB, PG, and AP: writing and reviewing. NI: conceptualization, writing, and reviewing. JH: conceptualization and reviewing. All authors contributed to the article and approved the submitted version.

## Funding

Philip Morris International is the sole sponsor of this study.

## Conflict of Interest

The authors are employees of Philip Morris International.

## Publisher's Note

All claims expressed in this article are solely those of the authors and do not necessarily represent those of their affiliated organizations, or those of the publisher, the editors and the reviewers. Any product that may be evaluated in this article, or claim that may be made by its manufacturer, is not guaranteed or endorsed by the publisher.
